# HPV和肺癌关系的研究进展

**DOI:** 10.3779/j.issn.1009-3419.2012.03.11

**Published:** 2012-03-20

**Authors:** 锋杰 郭, 亚光 范, 友林 乔, 清华 周

**Affiliations:** 1 300052 天津，天津市肺癌转移与肿瘤微环境重点实验室，天津市肺癌研究所，天津医科大学总医院 Tianjin Key Laboratory of Lung Cancer Metastasis and Tumor Microenvironment, Tianjin Lung Cancer Institute, Tianjin Medical University General Hospital, Tianjin 300052, China; 2 100021 北京，中国医学科学院北京协和医学院肿瘤医院肿瘤研究所 Cancer Institute, Peking Union Medical College and Chinese Academy

**Keywords:** 肺肿瘤, HPV, 进展, Lung neoplasms, HPV, Progress

## Abstract

肺癌是严重危害人类健康的恶性肿瘤之一，近50年来其发病率和死亡率呈现不断上升趋势，肺癌的发病率和死亡率在世界范围内均居各种癌症首位。肺癌的危险因素是多方面的。吸烟是其中一个重要风险因素，但不吸烟者（特别是女性）仍有一部分会患肺癌。许多研究认为人类乳头瘤病毒（human papillomavirus, HPV）是肺癌的危险因素。然而，HPV作为肺癌的风险因素，对其进行的全面认真的评估较少。由于检测方法、地区分布、样本量等存在差异造成HPV感染和肺癌的相关性研究结果也不尽相同。近年来，随着研究的不断深入，HPV与肺癌的关系日益受到重视。现将近年关于HPV和肺癌关系的研究进展作一简要综述。

## Abstract (en)

Lung cancer is one of malignant tumors harming human health. Over the past five decades, there is increasing morbidity and mortality of lung cancer which is the leading cause of cancer morbidity and mortality worldwide. Risk factors of lung cancer are versatile and smoking is among the important ones, but there are some non-smoking men, especially for women, some of whom developed lung cancer. Many studies showed that human papillomavirus (HPV) was the risk factor of lung cancer, however, which was less comprehensive or seriously estimated. The results of research on relationship between HPV infection and lung cancer are different because of the difference of detection methods, geographical distribution and sample size. Recently, the relationship of HPV and lung cancer is increasingly thought highly with deep study. Study advance of relationship between HPV and lung cancer in the recent years is briefly reviewed.

## 1 肺癌流行病学现状

我国是世界第一肺癌大国，每年大约有50万人死于肺癌，2008年卫生部公布的第三次全国居民死亡原因调查结果显示，肺癌已位居中国恶性肿瘤首位。肺癌的病因和发病机制迄今尚未明确。危险因素是指会增加人们患某种疾病机会的因素。一般认为肺癌的发病与以下因素有关，包括吸烟、环境污染、呼吸系统疾病、二手烟、职业致癌因素、室内氡污染等^[1-4]^。其中吸烟已被公认为是引起肺癌的最重要的危险因素。在欧美发达国家，肺癌的发生归因于吸烟的危险度在90%左右。20世纪80年代，上海市区男性肺癌归因于吸烟的危险度为70%-80%；女性肺癌约30%归因于吸烟与被动吸烟。在一项研究^[5]^中，49%的中国非吸烟女性的肺癌发生可归因于吸烟（主动和被动）、职业因素、室内氡污染及低水果摄入，但超过50%的非吸烟女性肺癌归因危险尚不确定。

尽管吸烟是肺癌发病的首要原因，但非吸烟者尤其是女性的肺癌发病近年来明显增加。至少15%的肺癌患者一生都不吸烟，而且这个数字在东亚女性中更高，尽管她们的吸烟率较低（低于4%的成年烟民），但中国女性肺癌发病率比许多西方国家的女性肺癌发病率还要高。亚洲女性的肺癌发病与其它种族或地区存在较大的差异，除室内燃煤污染外，包括人类乳头瘤病毒（human papillomavirus, HPV）在内的感染因素可能是重要因素^[6]^。

## 2 HPV

HPV属于乳多空病毒科的乳头瘤空泡病毒A属，是球形DNA病毒，能引起人体皮肤、粘膜的鳞状上皮增殖，包括了6个早期调控基因（E1, E2, E4, E5, E6, E7）和2个晚期基因（L1, L2）^[7]^。HPV可以选择性地感染鳞状上皮和粘膜上皮，而且特殊的HPV类型与鳞癌及宫颈、阴茎、肛门、阴道和外阴的异型增生有关。根据其与人类肿瘤的关系，HPV可分为高危型（HPV16, HPV18, HPV31, HPV33, HPV35）、中危型（HPV31, HPV33, HPV35）和低危型（HPV6, HPV11）^[8]^。高危型与宫颈癌、膀胱癌、大肠癌等关系密切，中危型与介于良性及恶性之间的病变有关，低危型与尖锐湿疣等良性皮肤疾病相关。大部分HPV感染并无症状，是一过性的，只有那些高危型HPV的持续感染才与肿瘤的发生有关。然而，持续性HPV感染者也只有少部分发生癌变。

## 3 HPV和肺癌

众所周知HPV可以导致宫颈癌，HPV感染与阴道、肛门和阴茎癌等外阴部癌症发生存在明显相关性^[9]^。最近的研究^[10]^表明，HPV感染也与头颈癌的发展有关。喉乳头状瘤病可累及气管和支气管，可扩散至远端支气管，引发气管、支气管的乳头状瘤甚至恶变，HPV也可播散到肺形成隐性感染，遇到可能的刺激后再引发肿瘤。HPV感染可能源于先天或后天的HPV感染。HPV感染要E6、E7蛋白首先通过1型干扰素抑制信号并降低多种干扰素诱导基因的表达，并通过E6、E7癌蛋白和细胞表面分子发生相互作用发挥功能。E6、E7蛋白可以通过降解抑癌基因*p53*、激活端粒酶、Rb活性等使细胞癌变^[11, 12]^。HPV16/18 E6在HPV DNA阳性的肺癌中可能通过抑制p53活性导致了HPV介导的肺癌发生^[13]^。

1979年和1980年，Syrjanen^[14, 15]^分别报道肺鳞癌周围的支气管上皮内有与生殖道湿疣相似的病变，指出湿疣型病变和宫颈癌相关而且要重视肺内类似病变，并发现30%的支气管鳞状上皮组织活检提示有HPV感染的形态学改变，进而提出了HPV可引起肺鳞癌发生的假说。1987年，Syrjanen^[16]^报道用HPV16、HPV11、HPV6、HPV18、HPV30型DNA混合型探针，对99例支气管鳞癌进行原位杂交检测HPV DNA序列检出率为5%，这是首次确定支气管鳞癌中HPV DNA的存在。从Syrjanen在肺癌中提出假说到确认HPV DNA的存在后，直到现在有一些研究^[17, 18]^肯定了肺癌和HPV感染有关，然后也有一些相反的观点。这可能是由于HPV检测方法、地区分布、样本量等存在差异造成的。

已发表的文献^[19]^报道，全世界肺癌中HPV平均感染率为24.5%，欧洲和美国分别为17%、15%，亚洲为35.7%。危险类型中较多的是高危型HPV16、HPV18、HPV31、HPV33，而低危型HPV6、HPV11也可被检出。组织类型中鳞癌较多，其它类型的非小细胞肺癌以及小细胞肺癌也有报道。

肺癌中HPV感染率存在着较大的地区差异。台湾的一项研究^[20]^发现，在肺癌病例和对照中HPV-16/18感染率分别为54.6%和26.7%。而在武汉进行的调查^[21]^发现，HPV-16/18在肺癌病例对照的感染率分别为27.7%、5.9%。日本的研究^[22]^报道称HPV-16/18在日本南部亚热带岛屿Okinawa的感染率为58%，在Niigata及本岛则较低分别为6.7%和23%。在西欧国家HPV感染和肺癌的相关性不太紧密。在法国，对218例新鲜冰冻肺癌组织进行的检测表明，仅4例样本是阳性的，并且E6 mRNA在这4例中也未检测到^[23]^。通过PCR对意大利的38例非小细胞肺癌样本进行HPV16、HPV18、HPV31的扩增检测发现，8例为阳性（21%），但是对照组的癌旁肺组织均为阴性^[24]^。在对拉丁美洲哥伦比亚、墨西哥、秘鲁三国收集的36例肺癌样本进行PCR和Southern blot检测发现，HPV阳性率为28%^[25]^。

在组织类型上，肺鳞癌被认为与HPV感染关系比较紧密，但是其它类型也有报道。在日本一肺鳞癌高发区Okinawa进行的研究^[26]^发现，HPV在腺癌的感染率为78.3%。Chiou等^[27]^在台湾进行的研究发现，HPV16在腺癌的感染率（55.6%）高于鳞癌（35.6%），同时，临床分期中Ⅰ期与Ⅱ期、Ⅲ期与Ⅳ期感染率分别为29.3%和54.6%（*P*=0.006）。一项法国研究^[28]^显示，HPV DNA在肺癌的阳性率为鳞癌2/18，腺癌为1/4，内分泌肿瘤为2/7，大细胞未分化癌为0/2。Yousem等^[29]^采用原位杂交技术和混合型探针（6/11、16/18和31/33/35）研究肺癌，20例中6例鳞癌发现HPV DNA序列（30%），6例大细胞未分化癌中有1例阳性，而32例腺癌、支气管肺泡癌、小细胞癌中无1例阳性，15%病例受感染肿瘤旁鳞状化区域显示类似HPV DNA表达。

HPV感染与非吸烟肺癌发生之间的关系是目前研究的热点问题。吸烟已成为肺癌的主要危险因素，但是大约有15%的肺癌患者不吸烟。尽管存在地区性差异，非吸烟肺癌患者以女性居多，组织分型上非吸烟患者腺癌多于鳞癌，家族聚集性在非吸烟患者中更突出。癌基因的突变在非吸烟肺癌中也较为突出，以*EGFR*突变和间变性淋巴瘤激酶激活较为常见^[30]^。当然如二手烟、激素、病毒等因素也会引起非吸烟性肺癌^[31]^。

Fei等^[21]^报道武汉人群HPV16、HPV18在非吸烟肺癌患者感染率高于吸烟者，分别为48.7%、29%和57.3%、20.6%（*P* < 0.001）。Cheng等^[20, 32]^报道台湾人群HPV16/18在非吸烟女性肺癌患者中的存在高于非吸烟男性肺癌患者，HPV6在非吸烟男性肺癌患者中的存在高于非吸烟女性肺癌患者（33.3% *vs* 11.1%, *P*=0.023），但是男性吸烟与不吸烟之间却没有明显差异（38.1% *vs* 33.3%），而且HPV6在吸烟肺癌患者的临床分期的早期感染率高于晚期，但在非吸烟患者中则不存在此差异。但也有相反的结论，如Lim等^[33]^研究结果否认了HPV在非吸烟肺癌患者中的高感染率。Park等^[34]^在对韩国人的调查中认为吸烟和HPV感染的存在没有相关性。因此，HPV感染与吸烟在肺癌的发生中是否存在交互作用需要进一步深入的评价。

近年来随着国内外对HPV感染与肺癌发生相关性研究的不断深入，研究结果表明两者之间存在密切联系。HPV感染是否确实与肺癌相关及归因危险研究可能对肺癌的病因学预防及控制提供进一步的帮助。但是，由于研究对象地域性、肿瘤标本取样差异、检测方法的差异，可能导致HPV检测结果存在较大的差异。因此，进一步探索HPV感染和肺癌的关系是非常必要的，但在研究中要考虑研究对象、方法差异所致的不同结果。

随着人们对HPV感染与肺癌相关性认识的提高和简便、快速、特异性、敏感性高的HPV检测新技术的发现和完善，HPV感染与肺癌相关性的问题必将得到正确的结论。
